# Ventricular septal necrosis after blunt chest trauma

**DOI:** 10.5249/jivr.v4i2.97

**Published:** 2012-07

**Authors:** Feridoun Sabzi, Mojtaba Niazi, Abdol Hamid Zokaei, Farzad Sahebjamee, Shahrzad Bazargan Hejazi, Alireza Ahmadi

**Affiliations:** ^a^Imam Ali Heart Center, Kermanshah University of Medical Sciences, Kermanshah, Iran.; ^*b*^Department of Psychiatry, College of Medicine, Charles Drew University of Medicine and Science & David Geffen School of Medicine, UCLA, Los Angeles, CA, USA.; ^*c*^ Imam Reza Hospital, Kermanshah, University of Medical Sciences, Kermanshah, Iran.; ^*d*^ Department of Public Health Sciences, Division of Social Medicine, Karolinska Institute, Stockholm, Sweden.

## Abstract

Ventricular Septal Defect (VSD) after blunt chest trauma is a very rare traumatic affection. We report here a case of blunt chest injury-related VSD and pseudoaneurysm. A 30-year old male truck driver was referred from a trauma center to our hospital seven days after a blunt chest trauma and rib fracture. The patient had severe pulmonary edema and echocardiography showed large VSD. Several mechanisms are involved in the pathogenesis of this affection including an acute compression of the heart muscle between the sternum and the spine, leading to excessive changes in the intrathoracic and most likely the intracardiac pressure after blunt chest injury. Traumatical patients with the same symptoms may be at risk of sudden death. Therefore, a high grade of suspicion is mandatory even without solid evidence of myocardial damage on the initial evaluation. In continue some hidden angles of this case was discussed. Given the prognostic implications of traumatic VSD with associated pseudoaneurysm, its detection has critical value for preventing its clinical sequelae.

## Introduction

Ventricular septal defect (VSD) after blunt chest trauma is a very rare traumatic affection.^1^ We report here a case of blunt chest injury-related VSD and pseudoaneurysm. Given the prognostic implications of traumatic VSD with associated peudoaneurysm^2-3^ immediate detection and treatments are extremely critical for the survival of the patient.

The true incidence of VSD following chest trauma is unknown in the literature. The autopsies of 207548 cases of nonpenetrating thoracic trauma related deaths due to car accidents identified VSD in 0.01 % of the cases, and in only 0.002% of cases the defects were isolated. Rupture of the free ventricular wall was the most common form of presentation among these cases.^4^ The incidence in clinical cases with severe chest trauma has been between 10% to 75%.^5^

In Brazil in 2002, there were 33288 deaths by car accidents according to data from DATASUS. Many of these individuals died secondarily to multiple trauma including head and chest trauma.^6^ In addition to blunt trauma, penetrating legions, coronary artery lesions and endocarditis may cause VSD.^7^

## Case Study

A 30-year old male truck driver was referred from a trauma center to our hospital seven days after a blunt chest trauma and rib fracture. Initial clinical examination in the emergency room revealed contusion of the chest wall and rib fracture. Chest X ray showed bilateral pulmonary edema, and auscultation of the heart revealed a III/IV holosystolic murmur with maximal intensity in the third left intercostal space, accompanied by a thrill palpable in the same area. Initial physical examinations revealed a moderate pericardial fluid and plural effusion. Blood analysis showed elevated cardiac enzymes: creatine phosphokinase was 2000 U/L (normal value < 60 and 400 U/L) CPK-MB was 100 Mg/L (Normal valve < 4 Mg/L) and troponin was as high as 40 Mg/L suggesting the presence of cardiac contusion. The echocardiogrophic evidences showed a large rupture of ventricular septum with a significant left to right shunt and apico-anterior pseudo aneurism. Due to the unstable condition of the patient, i.e., an unusual traumatic rupture of ventricular septum, and the hemodynamically unstable condition, immediate surgical repair was selected as our choice of treatment. 

Patient was transfer to operation room and anesthesia was inducted with Ketamine (100 mg), fentanyl (300 mic) and succinylcholine (100 mg). Anesthesia was maintained by isoflurane 0.4 %, fentanil 10 mic/min and Cisatracurium 2mg/hour. Cardiopulmonary bypass (CPB) was performed with mild hypothermia after aortic and bicaval cannulation. Exploration of the interventricular septum was first tried through the right atrium and tricuspid valve and a large antroapical VSD was seen. The VSD was repaired with Dacron patch with separate sutures.

After surgery, patient was transferred to ICU without extubation. The anesthesia drugs and muscle relaxation were not reversed. The postoperative echocardiography confirmed a small residual VSD and the postoperative ECG did not demonstrate any block or any other arrhythmia and the patient was discharged 20 days after the operation.

## Comment

The myocardial contusion is one of the most frequent lesion of the heart, often observed after blunt chest trauma and usually prognosis is good.^8^ However, sometimes Dressler syndrome develops from more serious blunt injures to the heart followed by the rupture of the ventricles or atria, which leads to instantaneous pericardial tamponade and death.^9^ Therefore, before hospitalization detailed physical and cardiac exam of the patient should be performed.^10^ Rupture of the ventricular septum is believed to occur due to compression of the chest wall while the ventricles are filled with blood and the heart valves are closed resulting in excessive intra ventricular pressure.^11^

Post traumatic VSD or free wall rupture is infrequent,^1,3^ and mostly becomes symptomatic after an injury event, although very few of the patients may remain asymptomatic during the initial exam^8^ and show gradual decompensation. Given the prognostic implications of traumatic VSD with associated pseudoaneurysm, its detection is of critical value for preventing possible clinical sequelae, which may include imminent death.^2-3^ Therefore, a high level of suspicion is mandatory even without solid evidence of myocardial damage during the initial evaluation.

The early closure of VSD is recommended, although some experts advise conservative approach for asymptomatic cases, because the margins of the defect become fibrous in time and this provides an easy and safe suture line.^12^ Whereas, when a large unrestrictive intraventricular communication exists, homodynamic decompensation develops more rapidly. In our case, the VSD was very large and caused a significant left to right shunt. This probably explains the patient’s homodynamic decompensation which was associated with sever congestive heart failure, (sever pitting edema, ascitis, pulmonary edema). One other complication after the treatment in our case was the development of left ventricular pseudoaneurysm as a result of contusion.

The mechanism of the myocardial contusion involves disruption of coronary blood flow or intramural hematoma.^13^ Our literature review of the successful repair of combined VSD and pseudo-aneurysm following blunt trauma only found five such cases.^14-18^ Once cardiac injury is suspected, structural cardiac damage should be overruled using echocardiography, the echocardiography should be repeated after several days, since delayed rupture is possible after blunt force trauma of.^9^

**Figure 1: Large Ventricular Septal Defect with diameter equal to 1.58 cm in upper septum, good Left Ventricular Ejection Fraction equal to %55-%60, large pericardial effusion:**
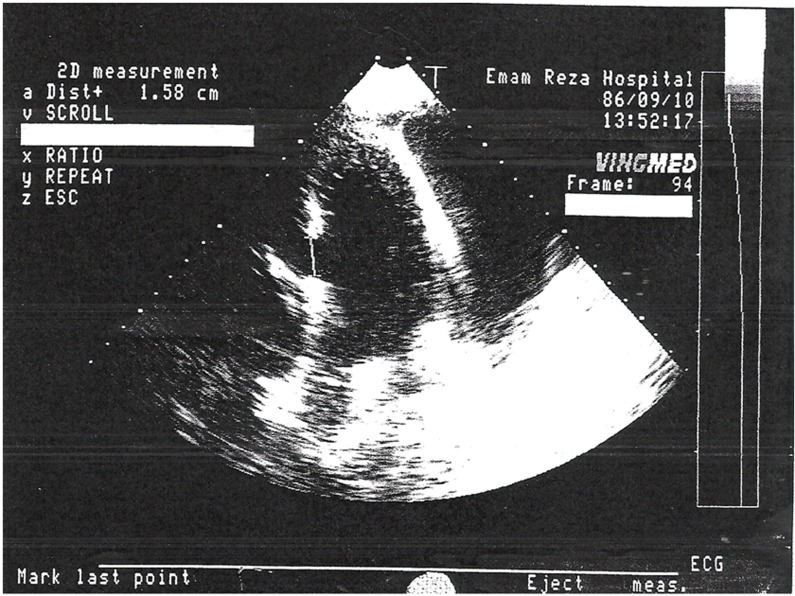


An autopsy report from Brazil indicated that 15% of fatal chest trauma victims died from myocardial contusion.^19^

Our patient was presented with an unstable condition and our management of hemodynamically unstable patient with a large septal rupture consists of; inotropic support, left ventricular afterload reduction by medical therapy as well as placement of an intra aortic balloon pump and urgent surgical closure of such defects. The VSD was 4×4 cm large and it was immediately decided to perform surgical closure of this defect.

The concomitant rupture of the ventricular septum and free wall as pseudoaneurysm appears to be rare.^20^ A search of the literature revealed six such cases of combined complications after blunt chest trauma. In our patient, post traumatic myocardial contusion was most likely the responsible mechanism for the formation of pseudoaneurysm during the following days of accident. A small residual VSD remained despite carful surgical repair, which is relatively asymptomatic and is managed medically.

In conclusion, we recommend the closure of the traumatic VSD in its early phase. After the stabilization of the homodynamic status the septal repair of VSD can be a safe method for such defects. It should be noted that the absence of myocardial damage immediately after the blunt chest trauma does not exclude presence of VSD risk. Therefore, initial clinical examination including thorough history and physical exams are highly recommended.^7,20,21^
